# Leiomyosarcoma of intravascular origin - a rare tumor entity: clinical pathological study of twelve cases

**DOI:** 10.1186/1477-7819-8-103

**Published:** 2010-11-22

**Authors:** Daniel J Tilkorn, Joerg Hauser, Andrej Ring, Ole Goertz, Ingo Stricker, Hans U Steinau, Cornelius Kuhnen

**Affiliations:** 1 Operative Reference Center for soft tissue sarcoma, BG University Hospital Bergmannsheil, Ruhr University Bochum, Germany; 2 Institute of Pathology, BG-University-Hospital Bergmannsheil, Ruhr-University, Bochum, Germany; 3 Institute of Pathology at the Clemenshospital Münster - Medical Center, Münster, Germany

## Abstract

**Background:**

Leiomysarcoma of intravascular origin is an exceedingly rare entity of malignant soft tissue tumors. They are most frequently encountered in the retroperitoneum arising from the inferior vena cava and are scarcely found to arise from vessels of the extremities. These tumors were analysed with particular reference to treatment outcome and prognosis. The aim of this article is to broaden the knowledge of the clinical course of this rare malignancy.

**Method:**

During 2000 and 2009 twelve patients were identified with an intravascular origin of a leiomyosarcoma. Details regarding the clinical course, follow-up and outcome were assessed with focus on patient survival, tumor relapse and metastases and treatment outcome. 3 year survival probability was calculated using Kaplan-Meier method.

**Results:**

Vascular leiomyosarcomas accounted for 0.7% of all malignant soft tissue tumors treated at our soft tissue sarcoma reference center. The mean follow up period was 38 months. Tumor relapse was encountered in six patients. 6 patients developed metastatic disease. The three year survival was 57%.

**Conclusion:**

Vascular leiomysarcoma is a rare but aggressive tumor entity with a high rate of local recurrence and metastasis.

## Background

Malignant soft tissue tumors account for < 1% of malignant tumors in adults [1], Leiomyosarcomas make up only < 5% of these rare soft tissue tumors [2].

Intraabdominal, retroperitoneal, cutaneous, subcutaneous and vascular growth patterns can be distinguished. Vascular leiomyosarcoma arises from major blood vessels and originates directly from the muscular wall of these vessels.

Up to 75% [3] arise from the retroperitoneal course of the inferior vena cava [4]. As with other soft tissue sarcomas clinical presentation may be delayed due to their deep origin. Presentation include palpable masses or intraluminal obstruction with signs of venous stases, thrombosis or embolism. Extracaval venous branches are a particularly rare source of vascular leiomyosarcomas and most frequently involve venous branches of the lower extremity [4]. Due to its rare incidence and the unusual clinical course the diagnosis and special treatment requirements are often challenging for the multidisciplinary team. We reviewed our experience with vascular leiomysarcomas with special reference to the clinical course and outcome. Differential diagnosis, clinical and pathological criteria will be discussed.

## Methods and Materials

A search of our prospectively updated soft tissue tumor database revealed 182 patients diagnosed with a leiomyosarcoma from 2000 to 2009 at the BG University Hospital Bergmannsheil. Twelve of these tumors had an intravascular origin. Patients' notes were evaluated and patients and their physicians were contacted for details regarding the clinical course, follow-up and outcome. Follow-up imagine was obtained for all patients and included chest X-ray or CT scan, abdominal ultrasound, CT or MRI scan of the tumor site.

Local recurrence was defined as tumor relapse at the primary tumor resection site and metastatic disease was defined as tumor growth at any other site. Results are presented as means and standard deviation. Three year survival was calculated using the Kaplan - Meier method.

Due to the small number of patients we refrained from further statistical analysis.

### Histology

Histological slides of the primary tumor were (re-) assessed by a pathologist* with expertise in soft tissue pathology from the Institute of Pathology, BG University Hospital Bergmannsheil. In all cases additional immunohistochemistry was performed to confirm the diagnosis. Second expert opinions from another pathologists were obtained in two cases. The primary diagnosis of an intravascular leiomyosarcoma of our institute was confirmed in both cases.

*The histopathological assessment by Prof. C.Kuhnen was carried out predominantly during his time at the Institute of Pathology, BG-University-Hospital "Bergmannsheil", Ruhr-University, Bochum, Germany

## Results

1613 patients with a malignant soft tissue tumor (MSTT) were treated at our institute between 2000 and 2009. 182 tumors (11% of all MSTT) were diagnosed as leiomyosarcoma. Twelve of these tumors were of intravascular origin. Intravascular leiomyosarcoma accounted for 0.7% of all MSTT or 6% of all leiomyosarcomata. The mean age at the time of diagnosis was 59 years (± 10.7). Nine patients were females and three were males. The mean follow-up time from the time of definite surgery of the primary tumor was 38 months (± 30.6). The three year survival was 57%. Due to the insidious onset of the disease and late clinical symptoms the time between onset of tumor growth and the definite diagnosis could not be assessed.

The site of tumor growth was the lower extremity in 67%, the upper extremity in 25% and the vena cava in 8%.

In 50% the disease initially presented with signs of venous stasis. Deep venous thrombosis (n = 1), thrombophlebitis of the long saphenous vein (n = 2) and acute pulmonary embolism (n = 1) occurred as initial symptoms of the malignancy.

67% of the tumors were subfascial and 25% epifascial. In 33% the tumor originated from the femoral vein. Mean tumor size was 7.4 cm in largest diameter (± 3.0).

17% of the tumors were T_I _and 83% were T_II_.

At primary diagnosis no metastatic disease was detected in any patient.

According to the Coindre classification of tumor grading for soft tissue tumors, the tumors were subdivided in high grade (42%), intermediate (42%) and low grade (16%). (Table 1)

**Table 1:** Data of the patient collective and clinical course of the tumor disease.

Patient	Age at the time of diag.	Localisation	Affected vessel	Size in cm	TNM	Comments	Thrombembolic event	Status
**1**	66	right upper extremity, supra-clavicula region	internal Jugular vein/sub-clavian vein	7.6 × 8 × 3.3	T2 N0 M0 G2	second expert opinion	n	DOD

**2**	74	left thigh	femoral vein	-	--	no diagnosis before primary surgery	n	DOD

**3**	77	Left upper extremity, wrist	accompaniing ulna vein	2.7×1.7×1.2	T1 N0 M0 G1	initial surgery for a suspected wrist ganglion revealed the diagnosis of a intravascular leiomyosarcoma	n	alive

**4**	52	right thigh	femoral vein	6.4 × 7 × 9	T2 N0 M0 G3	palpable tumor 4 month before diagnosis	n	DOD

**5**	55	left dorsum of the upper arm	subcutaneous Vein	7.8 × 4.5 × 5	T2 N0 M0 G3	palpable tumor 2 month before diagnosis	n	alive

**6**	64	right thigh	subcutaineous vein	2.5 × 2 × 1	T1 N0 M0 G2	palpable tumor 3-4 month before diagnosis; external primary tumor enucliation second expert opinion	n	alive

**7**	41	retro-peritoneum	inferior vena cava	8.8 × 6 × 5	T2 N0 M0 G1	primary histology Pathol. BG-Uni. Bergmannsheil Bochum; Second expert opinion Oncological resection Dep. Visceral Surg. Uni Bochum	n	alive

**8**	56	right thigh	external iliac vein, femoral vein	10.1 × 7.5 × 5	T2 N0 M0 G2	3 tumor free lymph nodes in primary histology Oncological resection Dep. Visceral Surg. Uni Bochum	deep vein throm-bosis	alive

**9**	61	left thigh	femoral vein	2.3 × 2 × 6	T2 N0 M0 G2	-	n	DOD

**10**	59	left thigh	femoral vein	9.6 × 6 × 4	T2 N0 M0 G3	Histology was reassessed after transferral due to a tumor relapse	pulmonary embo-lism	alive

**11**	44	left thigh	great saphenous vein	12 × 4 × 3.6	T2 N0 M1 (lung, liver) G3	palpable small tumor, suspected to be a lipoma two years prior to diagnosis	thrombophlebitis of the saphenous vein	alive

**12**	56	left lower leg	great sahpenous vein	5 × 4 × 1	T2N0 M1 (pulmo) G3	crossectomy	thrombophlebitis preop., paget shrotter syn-drome postop.	alive

Primary diagnosis was provided by incisional biopsy in two cases and punch biopsy in a further two cases prior to surgical ablation. Five patients were resected with microscopically positive margins at other institutes and were referred to our center for definitive ablation. One patient presented with the clinical symptoms of a wrist ganglion. After the initial tumor resection the histological assessment revealed a vascular leiomyosarcoma. The definite surgical intervention required a resection of the ulnar artery and accompanying vena comitantes. Two patients were transferred to our center after tumor relapse. One of them initially underwent vascular bypass surgery for a suspected malignant fibrous histiocytoma (N^O.^ 2). The initial incorrect pathological assessment was benign. Tumor relapse led to the diagnosis of aleiomysarcoma. At that time the tumor surrounded the vascular prosthesis and hip implant. Amputation was discussed but refused by the patient. Palliative radiation therapy was initiated and the patient died five months later. In the other patient (N^O.^ 12) during stripping of the long saphenous vein a tumor mass was encountered and the surgical specimen was sent for histology which demonstrated a leiomyosarcoma. The tumor was resected with positive margins. Despite adjuvant radiation therapy the tumor relapsed. The patient was then admitted to an oncology department where he received adjuvant chemotherapy before being transferred to our center for intervention. Surgery with curative intent required an extended hip amputation. At present there is no evidence of metastatic disease after 14 months.

Three patients had femoral vessel resection and a subsequent arterial bypass.

Patient N^O.^ 7 required partial resection of the vessel wall of the inferior vena cava. In N^O.^ 11&12 the tumor bearing segment of the long saphenous vein was resected. The ulnar artery and vein were resected in one patient.

Six patients received adjuvant radiation therapy of the primary tumor bed with a dose of 60 - 72 Gy. Three (N^O.^ 2,4&9) of these patients died of the disease, one of them (N^O.^ 2) received radiation therapy with palliative intention and one (N^O.^ 4) had microscopically positive resection margins after surgical ablation.

Six patients (N^O.^1;2;,4;9;11 & 12) developed pulmonary metastasis, one patient also suffered from bone metastasis (N^O.^9), another one (N^O.^11) had additional liver metastasis. Five of these patients (N^O.^1;2;9;11 & 12) also developed recurrent disease. In further two patients (N^O.^1;2) pulmonary metastasis was suspected approximately 6 (N^O.^1) and 4 (N^O.^2) months after the definite operation but according to the patients' wishes no tests were done to confirm the metastatic disease. All but two (N^O.^11 & 12) patients with metastatic disease died (N^O.^1 eighteen months N^O.^2 six months N^O^4 ten months and N^O^9 twenty eight months after the final diagnosis was made). One patient received neoadjuvant chemotherapy (N^O.^9).

Five of these patients sought palliative chemotherapy (N^O.^ 1, 4,10,11 & 12). Table 2

**Table 2:** Follow up data of the treatment related course

N^O^.	Age/sex	Follow up period in month	Untreated tumor growth	Definite proce-dure	Adjuvant radiation-therapy	Chemo-therapy	Local recurrence	Meta-stasis	Time to death
**1**	66/f	18	-	R0	-	adjuvant	5 months	lung	18 months

**2**	74/f	15	2years ?	R1	60Gy	-	tumor-progression	lung?	15 months

**3**	77/m	84	-	R0	-	-	-	-	alive

**4**	52/f	10	2 month	R1	60Gy	-	-	lung	10 months

**5**	55/f	82	-	R0	60Gy	adjuvant	45 months	-	alive

**6**	64/m	78	4 month	R0	-	-	-	-	alive

**7**	41/f	64	-	R0	-	-	-	-	alive

**8**	56/f	43	-	R0	60Gy	-	-	-	alive

**9**	61/f	28	-	R0	60Gy	-	25 months	lung/bone	28 months

**10**	59/m	7	immediate	R1	-	neo-adjuvant	2 months	-	alive

**11**	44/f	14	2 years	R0	-	adjuvant	12 months	lung/liver	alive

**12**	56/f	14	-	R0	72Gy	adjuvant	10 months	lung	alive

### Macroscopic appearance

**Figure 1:**
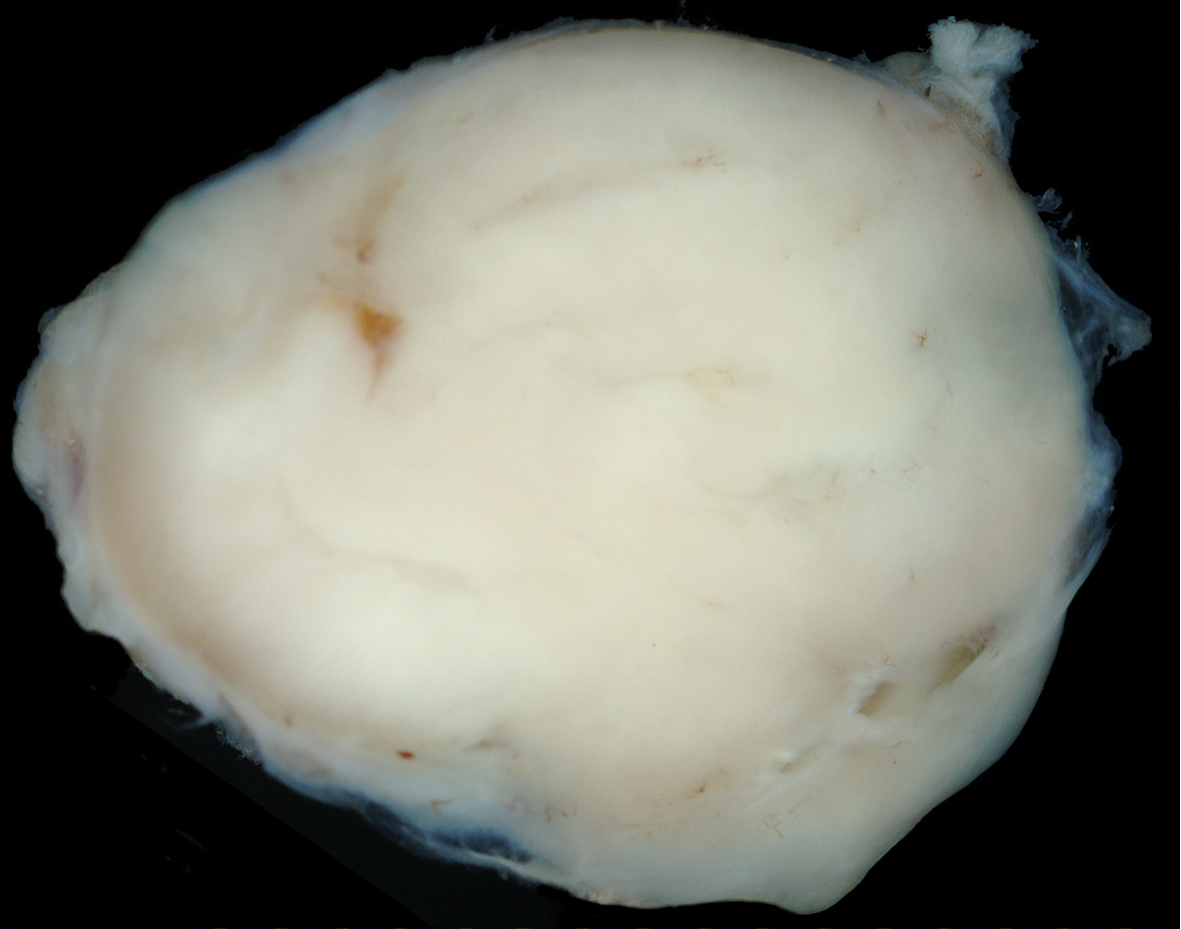
**Macroscopic appearance of a leiomysarcoma specimen after tumor resection at the wrist**.

In all cases tumor growth in close proximity to vascular structures was present. On resection of the tumor bearing vessels the tumors were found to originate from structures of the vessels walls and to exhibit an intravascular tumor sprout. Figure 1

### Microscopic appearance of the tumor

**Figure 2:**
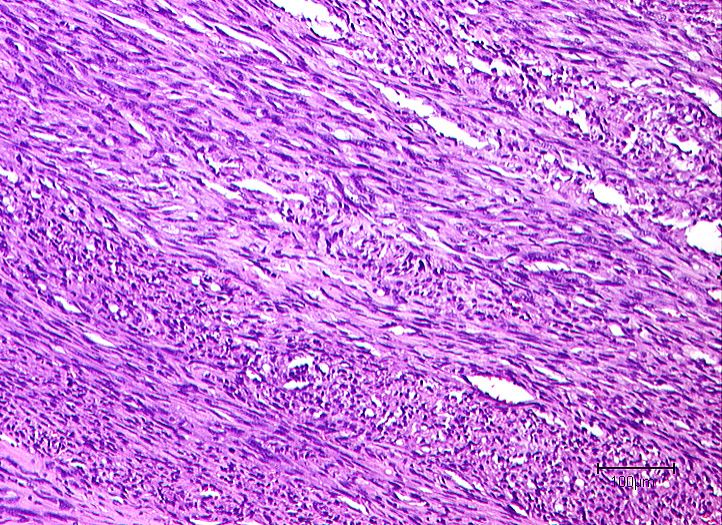
**Histological appearance of a leiomyosarcoma after resection of a tumor in the subclavicular region**. Notice the "cigar shaped" configuration of tumor cell nuclei with nuclear atypia (H&E staining).

Histologically a spindle cell, mesenchymal neoplasm was found in all cases associated with the media of the vessel wall, which was assessed as site of tumor origin. The tumor cells were characteristically forming various fascicles and showed an eosinophilic cytoplasm with cigar shaped nuclei. Figure 2

**Figure 3:**
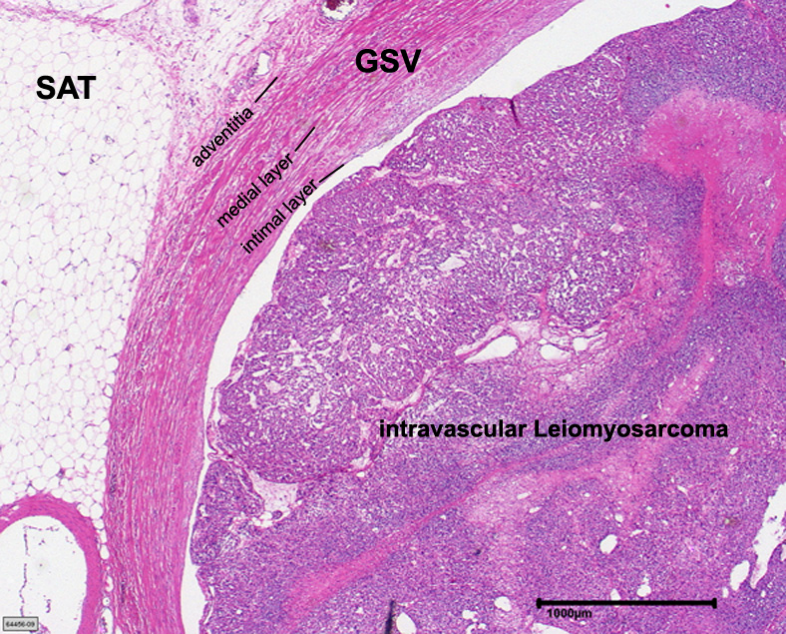
**Intraluminal tumour growth of a Leiomyosarcoma originating from great saphenous vein (GSV) (H&E-staining)**. The subcutaneous adipose tissue is labelled SAT.

The neoplasm derived from the media of the vessel wall and disrupted the existing vascular architecture. Immunohistochemically the majority of tumor cells displayed a positive reaction for smooth muscle actin and desmin, confirming smooth muscle differentiation of the tumor. Figure 3

## Discussion

Vascular leiomyosarcomata represent only a small proportion of soft tissue leiomyosarcomata [4]. All publications in the literature are of small clinical series or case reports. 11% of the patients treated for a malignant soft tissue tumor at the BG University Hospital Bergmannsheil presented with the leiomyosarcoma. The vascular leiomyosarcomas accounted for 6% of these tumors [5,6] Svarvar et al. recognised an even sex distribution in his study of 225 patients with all types of leiomyosarcoma [6].

In the current series nine patients with a vascular leiomysarcoma were female and three male. In the series of patients with vascular leiomysarcoma from Berlin et al. 5 were males and 1 female [7], in Abed et al.´s group 9 were female and 7 male [5] and in Dzsinich et al.'s series 12 were female and 1 male [8].

These tumors mainly originate from the media of venous vessel walls, with rare exceptions in which they derive from the arterial vessel structures [9]. The tumor is encountered in the retroperitoneal course of the inferior vena cava in 75% of all intravascular leiomyosarcomata [3]. The patients in our study are mainly extremity soft tissue tumors.

Venous obstruction and a palpable tumor mass were the most common symptoms. Deep venous thrombosis and even pulmonary embolism may be the initial clinical symptoms and can camouflage the clinical manifestation disease [10].

Leiomyosarcomas arising form smaller vessels are less frequent and may present primarily as nerve compression syndrome [4]. These tumors often protrude through small lumina of adjacent venous branches [7]. One of the patients presented with symptoms of arterial occlusion requiring vascular bypass surgery. Unfortunately in this particular case the delayed diagnosis in conjunction with an incorrect pathological assessment delayed the surgical ablation which permitted tumor progression. At this late time point to the patient refused surgical ablation and instead sought palliative radiation therapy.

Venous branches of the lower extremity as well as the azygos vein have been described as unusual sites of manifestation of intravascular leiomyosarcomata in the current literature [11,12]. Tumors localised in the upper extremity or the head and neck region are rare exemptions with reports in the literature limited to single cases [3,13-15]. In our intravascular leiomyosarcomas of the extremities there were four patients with a tumor origin of the femoral vein, further two cases of larger subcutaneous vein branches of the thigh, two of the long saphenous vein and most interestingly, three patients where the tumor was found in the upper extremity. Similar to our findings Berlin et al. [7] and Abed et al. [5] described the majority of these vascular leiomyosarcomas as arising from the lower extremity. In the combined studies of Abed et al. [5]and Dzsinich et al. [8] and Berlin et al. only one tumor was found in the upper extremity [7].

It has been indicated that small vein structures are the predominant source of the rare intravascular leiomyosarcomata of the deep somatic soft tissue [16].

The clinical picture may either result form the tumor growth itself or be related to the vascular occlusion manifesting as venous stases or thrombosis. A wide range of differential diagnosis may lead to clinical symptoms of venous stasis in the upper and lower extremity for example lung cancer, lymphomas, post thrombotic syndrome [17]. Intravascular neoplasms result in stasis of the blood flow by intraluminal obstruction [18,19]. CT scans, MRI, and angiograms [7,20] demonstrate filling defects in accordance with the clinical signs of vascular compression are valuable diagnostic measures that will facilitate the operative planning when suspecting an intravascular leiomyosarcoma. Particularly MRI can assist in differentiating intravascular tumor growth from thrombosis. In contrast to the intravascular tumor a thrombus commonly presents with a high signal intensity both in the T1 and T2 weighted images whereas the leiomyosarcoma appears as a homogenous tumor with an intermediate signal intensity on T1 - weighted imaging [21]. Moreover CT and MRI will not in all cases allow for an exact image of the endovascular tumor component [19].

An MRI was obtained in all of the presented patients prior to surgical ablation. Two subcutaneous tumors were ablated without imaging of the tumor prior to transfer to our center. The MRI in these patients was conducted before definitive surgery. A preoperative angiogram even though desirable was performed only in the minority of cases. Tumors of the venous vessel wall may present with an intraluminal growth pattern or may extent from the tunica media and infiltrate the surrounding soft tissue [13]. In thin veins especially extension into the perivascular soft tissue may occur early [4].

As vascular leiomyosarcomata are commonly composed of an intraluminal as well as an extravascular tumor component [7] after biopsy diagnosis of a soft tissue leiomyosarcoma the rare possibility of a primary intravascular tumor growth has to be suspected since it may influence the surgical strategy. Primary intravascular tumor growth may necessitate vascular dissection and resection of the tumor bearing course over a longer distance far from the palpable tumor mass due to intraluminal tumor extension [7,14]. The extravascular component of the sarcomata sometimes requires the resection and reconstruction of the adjacent artery as in 33% of the assessed patients.

In these cases both pre- and postoperative pulmonary microthrombembolism are frequent complications especially for tumors of the pulmonary artery [22]. The lung also is the most common site of distant metastasis [16]. Leiomyosarcomata are malignant tumors of mesenchymal origin with a differention towards smooth muscle morphology. The histological appearance is composed of spindle shaped cells with eosinophilic cytoplasm with muscular striation and cigar shaped rounded nuclei. Immunohistochemical staining for contractile fibers proteins such as actin, desmin as well as h-caldesmon can verify the diagnosis. Standard H&E staining and immunohistochemical staining for smooth muscle markers was performed in all of the cases of this series to confirm the diagnosis.

The differential diagnosis embraces the spectrum of spindle cell shaped neoplasms such as benign and malignant tumors of the nerve sheaths, myofibroblastic tumors (myofibromatosis, fibromatosis, myofibroblastic sarcoma), synovial sarcoma, fibrosarcoma and NOS sarcoma [23].

Intimal sarcoma, malignant mesenchymal tumors of the large arteries which originate from the intimal layer of the vessel wall [24] and the very rare intravascular angiosarcoma belong to the differential diagnosis of malignant intravascular tumors [25].

Surgical ablation with clear margins is the therapy of choice [5-7].

Vascular leiomyosarcomata are associated with aggressive tumor growth, poorer prognosis and earlier onset of metastatases compared to other soft tissue tumors [26]. The high rate of local recurrence and pulmonary metastases seen in the present study confirm these findings. Adjuvant radiation therapy may aid in local tumor control in the case of incomplete tumor resection or higher tumor grade. Tumor size and localization are of prognostic value [13]. Retroperitoneal leiomyosarcoma has similar out come and prognosis to leiomyosarcoma of the extremities [27].

Intravascular growth is associated with early pulmonary metastasis [16]. The three year survival of 57% in the presented study is consistant with the literature. [5-7] We recommend to include all patients with leiomyosarcomas in an international database to gain epidemiological data and improve the treatment of these rare tumors.

## Conclusion

Leiomyosarcomas rarely arise from blood vessel walls. The clinical presentation is misleading and thrombembolic events may be the first symptoms. Intravascular spread may complicate the surgical resection, rendering it difficult to obtain clear margins. The extravenous tumor component may necessitate resection of the concomitant artery or vein which requires vascular reconstruction. Early hematogenous metastasis and a high rate of local recurrence compromise the prognosis of the disease.

## Competing interests

The authors declare that they have no competing interests.

## Authors' contributions

DT conceptualized the study, gathered the data and wrote the manuscript. JH drafted and revised the manuscript. AR gathered the clinical data and assisted with interpretation of the data. OG reviewed the literature and assisted with the interpretation of the data. IS assessed the histological specimens. HS conceptualized and supervised the process of data gathering and revised the final. CK assessed the histological specimens, aided drafting and manuscript revision. All authors read and approved the final manuscript.

## References

[B1] Issels R Manual Knochentumoren und Weichteilsarkome 2004 4 München: W Zuckerschwerdt Verlag

[B2] Shimoda HOK Otani S Hakozaki H Yoshimura T Okazaki HNS Tomita S Oka T Kawasaki T Mori N Vascular leiomyosarcoma arising from the inferior vena cava diagnosed by intraluminal biopsy Virchows Arch 1998 8 97 100 10.1007/s004280050223 9692833

[B3] Varela-Duran J Oliva H Rosai J Vascular leiomyosarcoma: the malignant counterpart of vascular leiomyoma Cancer 1979 8 1684 1691 10.1002/1097-0142(197911)44:5<1684::AID-CNCR2820440523>3.0.CO;2-I 498039

[B4] Weiss SWGJ Weiss SW, Goldblum JR Leiomysarcoma (Hrsg) Enzinger and Weiss's soft tissue tumors 2001 4 Mosby, StLouis Baltimore Berlin 727 748

[B5] Abed R Abudu A Grimer RJ Tillman RM Carter SR Jeys L Leiomyosarcomas of vascular origin in the extremity Sarcoma 2009 8 385164 10.1155/2009/385164 19587823 PMC2705766

[B6] Svarvar C Bohling T Berlin O Gustafson P Folleras G Bjerkehagen B Domanski HA Sundby Hall K Tukiainen E Blomqvist C Clinical course of nonvisceral soft tissue leiomyosarcoma in 225 patients from the Scandinavian Sarcoma Group Cancer 2007 8 282 291 10.1002/cncr.22395 17154171

[B7] Berlin O Stener B Kindblom LG Angervall L Leiomyosarcomas of venous origin in the extremities. A correlated clinical, roentgenologic, and morphologic study with diagnostic and surgical implications Cancer 1984 8 2147 2159 10.1002/1097-0142(19841115)54:10<2147::AID-CNCR2820541015>3.0.CO;2-9 6488138

[B8] Dzsinich C Gloviczki P van Heerden JA Nagorney DM Pairolero PC Johnson CM Hallett JW Jr Bower TC Cherry KJ Jr Primary venous leiomyosarcoma: a rare but lethal disease J Vasc Surg 1992 8 595 603 10.1067/mva.1992.34346 1560548

[B9] Perl L Ein Fall vom Sarkom der Vena cava inferior Virchows Arch 1871 8 378 385 10.1007/BF01957198

[B10] Subramaniam MM Martinez-Rodriguez M Navarro S Rosaleny JG Bosch AL Primary intravascular myxoid leiomyosarcoma of the femoral vein presenting clinically as deep vein thrombosis: a case report Virchows Arch 2007 8 235 237 10.1007/s00428-006-0322-2 17109153

[B11] Dasika U Shariati N Brown JM Resection of a leiomyosarcoma of the azygos vein Ann Thorac Surg 1998 8 1405 10.1016/S0003-4975(98)00718-8 9800844

[B12] Kevorkian J Cento DP Leiomyosarcoma of large arteries and veins Surgery 1973 8 390 400 4687797

[B13] Leu HJ Makek M Intramural venous leiomyosarcomas Cancer 1986 8 1395 1400 10.1002/1097-0142(19860401)57:7<1395::AID-CNCR2820570726>3.0.CO;2-O 3081244

[B14] Tovar-Martin E Tovar-Pardo AE Marini M Pimentel Y Rois JM Intraluminal leiomyosarcoma of the superior vena cava: a cause of superior vena cava syndrome J Cardiovasc Surg (Torino) 1997 8 33 35 9128119

[B15] Tilkorn DJ Lehnhardt M Hauser J Daigeler A Hebebrand D Mentzel T Steinau HU Kuhnen C Intravascular leiomyosarcoma of the brachiocephalic region -- report of an unusual tumour localisation: case report and review of the literature World J Surg Oncol 2008 8 113 10.1186/1477-7819-6-113 18954426 PMC2583979

[B16] Farshid G Pradhan M Goldblum J Weiss SW Leiomyosarcoma of somatic soft tissues: a tumor of vascular origin with multivariate analysis of outcome in 42 cases Am J Surg Pathol 2002 8 14 24 10.1097/00000478-200201000-00002 11756765

[B17] Puleo JG Clarke-Pearson DL Smith EB Barnard DE Creasman WT Superior vena cava syndrome associated with gynecologic malignancy Gynecol Oncol 1986 8 59 64 10.1016/0090-8258(86)90116-2 3943753

[B18] Weiss KS Zidar BL Wang S Magovern GJ Sr Raju RN Lupetin AR Shackney SE Simon SR Singh M Pugh RP Radiation-induced leiomyosarcoma of the great vessels presenting as superior vena cava syndrome Cancer 1987 8 1238 1242 10.1002/1097-0142(19870915)60:6<1238::AID-CNCR2820600613>3.0.CO;2-V 3304612

[B19] Izzillo R Qanadli SD Staroz F Dubourg O Laborde F Raguin G Lacombe P Leiomyosarcoma of the superior vena cava: diagnosis by endovascular biopsy J Radiol 2000 8 632 635 10844341

[B20] Deweese JA Terry R Schwartz SI Leiomyoma of the greater saphenous vein with preoperative localization by phlebography Ann Surg 1958 8 859 861 10.1097/00000658-195811000-00027 13595550 PMC1450898

[B21] Blum U Wildanger G Windfuhr M Laubenberger J Freudenberg N Munzar T Preoperative CT and MR imaging of inferior vena cava leiomyosarcoma Eur J Radiol 1995 8 23 27 10.1016/0720-048X(95)00608-S 7556247

[B22] Theile A "Walking pneumonia" in primary sarcoma of the pulmonary artery Pathologe 1996 8 231 234 10.1007/s002920050162 8710797

[B23] Mentzel TK Myofibroblastaere Tumoren. Kurzgefasste Uebersicht zur Klinik. Diagnose und Differentialdiagnose Pathologe 1998 8 176 186 10.1007/s002920050271 9648142

[B24] Bode-Lesniewska BKP Intimal sarcoma Fletcher CDM Unni KK Mertens (Hrsg) World Health Organization classification of tumours Pathology and genetics of soft tissue and bone IARC Press 2002 223 224

[B25] Hottenrott G Mentzel T Peters A Schroder A Katenkamp D Intravascular ("intimal") epithelioid angiosarcoma: clinicopathological and immunohistochemical analysis of three cases Virchows Arch 1999 8 473 478 10.1007/s004280050430 10592050

[B26] Gow CH Liaw YS Chang YL Chang YC Yang RS Primary vascular leiomyosarcoma of the femoral vein leading to metastases of scalp and lungs Clin Oncol (R Coll Radiol) 2005 8 201 15901009 10.1016/j.clon.2005.01.004

[B27] Hines OJ Nelson S Quinones-Baldrich WJ Eilber FR Leiomyosarcoma of the inferior vena cava: prognosis and comparison with leiomyosarcoma of other anatomic sites Cancer 1999 8 1077 1083 10.1002/(SICI)1097-0142(19990301)85:5<1077::AID-CNCR10>3.0.CO;2-0 10091791

